# Impact of Polyhexanide Care Bundle on Surgical Site Infections in Paediatric and Neonatal Cardiac Surgery: A Propensity Score‐Matched Retrospective Cohort Study

**DOI:** 10.1111/iwj.70710

**Published:** 2025-06-12

**Authors:** Gianluca Castiello, Giuseppe Caravella, Greta Ghizzardi, Gianluca Conte, Arianna Magon, Tiziana Fiorini, Laurenzia Ferraris, Simona De Vecchi, Veronica Calorenne, Andreea Alina Andronache, Alessandro Varrica, Alessandro Giamberti, Antonio Saracino, Rosario Caruso

**Affiliations:** ^1^ Intensive Care Unit IRCCS Policlinico San Donato Milan Italy; ^2^ Pharmacy Service IRCCS Policlinico San Donato Milan Italy; ^3^ Health Professions Directorate Bachelor in Nursing Course, ASST Lodi Lodi Italy; ^4^ Health Professions Research and Development Unit IRCCS Policlinico San Donato Milan Italy; ^5^ Health Professions Directorate IRCCS Policlinico San Donato Milan Italy; ^6^ Hospital Infection Control Committee IRCCS Policlinico San Donato Milan Italy; ^7^ Paediatric Care Unit IRCCS Policlinico San Donato Milan Italy; ^8^ Paediatric and Adult Congenital Heart Centre IRCCS Policlinico San Donato Milan Italy; ^9^ Department of Congenital Cardiac Surgery IRCCS Policlinico San Donato Milan Italy; ^10^ Clinical Research Service IRCCS Policlinico San Donato Milan Italy; ^11^ Department of Biomedical Sciences for Health University of Milan Milan Italy

**Keywords:** cardiac surgery, infection control, paediatric surgery, polyhexanide, surgical site infection

## Abstract

The primary aim of this study was to evaluate the impact of the polyhexamethylene biguanide (PHMB) care bundle on the occurrence rates of surgical site infections (SSIs) in paediatric and neonatal cardiac surgery, addressing a critical gap in paediatric‐specific infection prevention protocols. A retrospective cohort study included patients under 18 years old who underwent cardiac surgery at IRCCS Policlinico San Donato. Cohort A (*n* = 117) received the PHMB care bundle from April to December 2023, while Cohort B (*n* = 801) received conventional care from September 2020 to March 2023. The 1:1 propensity score matching was used to balance covariates between cohorts, resulting in two comparable cohorts (Cohort A = 114 patients and Cohort B = 112). The study found a significant reduction in SSIs among patients receiving the PHMB care bundle compared with those receiving conventional care (1.8% vs. 7.1%, *p* = 0.048). The comprehensive nature of the PHMB care bundle, including educational programs, preoperative and postoperative antimicrobial treatments, and consistent application of best practices, was instrumental in achieving these outcomes. Implementing antimicrobial care bundles could significantly reduce SSIs in paediatric cardiac surgery. Future research is needed to refine the tested bundle with prospective approaches.

**Summary**

- Successful infection prevention in paediatric cardiac surgery requires a comprehensive and multifaceted approach to address the unique vulnerabilities of this patient population.
- This study aimed to evaluate the impact of the PHMB care bundle on reducing SSIs in paediatric and neonatal cardiac surgery.
- This retrospective cohort study included patients under 18 years who underwent cardiac surgery, with 114 patients receiving the PHMB care bundle and 112 receiving conventional care after a 1:1 propensity score matching procedure.
- Implementing the PHMB care bundle reduced SSIs from 7.1% in the conventional care cohort to 1.8% in the PHMB care bundle cohort.

## 1 Introduction

Congenital heart diseases (CHDs) stand as a significant public health concern, representing approximately one‐third of all major congenital anomalies in Europe [1]. These structural abnormalities of the heart, present from birth, encompass a wide spectrum of conditions that profoundly affect the lives of paediatric patients [2, 3]. The complexity of CHDs necessitates surgical interventions, which, while life‐saving, are accompanied by substantial risks [3, 4]. Among these, surgical site infections (SSIs) emerge as a critical complication, further burdening this vulnerable population [5, 6].

SSIs, defined as infections occurring at or near a surgical incision within 30 days post‐operation or up to a year in the presence of implants, impose severe clinical and economic repercussions [5, 7, 8]. These infections are categorised into three distinct types based on the depth and location of the infection: superficial incisional, deep incisional and organ/space SSIs [9]. Superficial incisional SSIs involve the skin and subcutaneous tissue around the incision, presenting symptoms such as redness, warmth and drainage. Deep incisional SSIs extend deeper into the muscle and surrounding tissues, often requiring more aggressive intervention. Organ/space SSIs occur in any part of the anatomy that was opened or manipulated during the surgical procedure, posing significant challenges for diagnosis and treatment [5, 7, 8]. The pathogens responsible for SSIs typically originate from the patient's endogenous flora [10, 11]. *Staphylococcus aureus* , including methicillin‐resistant *Staphylococcus aureus* (MRSA), is the most frequently isolated organism [12]. Other common pathogens include coagulase‐negative staphylococci, Enterococcus species and *Escherichia coli* . These microorganisms can be introduced into the surgical site during the procedure or proliferate in the postoperative period, leading to infection [13, 14]. SSI diagnosis is based on a combination of clinical examination, laboratory tests and imaging studies, necessitating a comprehensive approach to identify and manage these infections effectively [15].

SSIs significantly contribute to postoperative morbidity and mortality, extend hospital stays and escalate healthcare costs [10]. The multifaceted impact of SSIs underscores their importance in paediatric cardiac surgery, where the stakes are extraordinarily high. The complexity of paediatric cardiac procedures, coupled with the fragile health of these young patients, creates a high‐risk environment for infections [7, 8, 10]. Inconsistent adherence to infection prevention protocols, including differences in preoperative skin preparation, use of prophylactic antibiotics and postoperative wound care, further contributes to varying SSI rates [6]. Consequently, the prevention and management of SSIs remain pivotal in enhancing surgical outcomes and overall patient quality of life [10].

In Italy, SSI rates from 2018 to 2020, collected by the European Center for Disease Prevention and Control (ECDC), show variability by surgery type and complexity. For example, cardiac surgeries had a relatively low SSI rate of 0.9% (95% CI: 0.6%–1.3%), while open abdominal procedures were higher at a pooled prevalence of 1.6% (95% CI: 1.2%–2.1%), with open colon surgeries reporting rates as high as 9.5% (95% CI: 9.2%–9.8%) [16]. Additionally, Italy's pan‐national SSI surveillance program reveals that in paediatric cardiac surgery, SSI rates exceed those seen in general cardiac procedures, reaching rates over 7% in more vulnerable groups, such as malnourished patients [17]. These epidemiological data are enriched by specific highlights derived from the general literature on paediatric cardiac surgeries, where SSI rates of 2.8% were recorded, with more frail subgroups (e.g., patients with malnutrition) showing SSI rates > 7% [13]. These data emphasise that procedural complexity and patient‐specific risks like nutritional status are key factors in SSI rates [13]. Infection prevention strategies may benefit from tailored approaches addressing these risks, particularly in complex and paediatric surgical settings, where SSIs could critically affect outcomes [18].

However, the lack of standardised, evidence‐based guidelines tailored specifically for paediatric cardiac surgery is a significant issue [11, 12, 13]. General infection control measures often do not address the unique needs of paediatric patients, resulting in variations in clinical practice and outcomes. In this context, polyhexamethylene biguanide (PHMB) emerges as a promising preventive solution due to its broad‐spectrum antimicrobial properties [19]. PHMB, a synthetic polymer belonging to the biguanide class, is known for its efficacy against a wide range of microorganisms, including bacteria, fungi and some viruses [19]. In this regard, a recent meta‐analysis has highlighted the potential of PHMB in reducing postoperative infections. A comprehensive meta‐analysis encompassing nine randomised controlled trials revealed a significant association between the use of PHMB and a reduction in the incidence of postoperative infections, with a notable decrease in infection rates (log Peto's odds ratio [OR], −0.890; 95% confidence interval [CI], −1.411 to −0.369; *I* ^2^ = 41.89%) [19].

Despite the promising potential of PHMB in reducing postoperative infections, there remains a notable gap in the literature regarding its specific application within structured care bundles for paediatric cardiac surgery [11, 12, 13, 14]. Current infection prevention protocols often lack the comprehensive inclusion of antimicrobial agents like PHMB because the evidence for such antimicrobial preoperative bundles remains limited. This gap is particularly concerning given the high stakes involved in paediatric cardiac surgeries, where SSIs can lead to severe complications and increased healthcare burdens. Applying PHMB within a structured care bundle could help address the gaps in current infection prevention protocols referred to paediatric cardiac surgery [20], even if the available evidence calls for empirical research to test the relationships of PHMB‐based care bundle and SSI rates within the framework of antimicrobial stewardship programs [7, 8, 11, 12]. For these reasons, the primary aim of this retrospective cohort study is to evaluate the impact of the PHMB care bundle on the occurrence rates of SSIs in the postoperative period of neonatal and paediatric cardiac surgery. Therefore, this study seeks to provide real‐world evidence regarding the potential impacts of PHMB in preventing SSIs by comparing SSI rates between patients who received a PHMB care bundle and those who received conventional care.

## 2 Material and Methods

### 2.1 Design

This study employs a propensity score‐matched, retrospective cohort design to evaluate the impact of the PHMB care bundle on the occurrence rates of SSIs in neonatal and paediatric cardiac surgery. The study was meticulously reported following the Strengthening the Reporting of Observational Studies in Epidemiology (STROBE) checklist to ensure comprehensive and transparent reporting of observational research [21]. Additionally, this study received ethical approval from the Italian National Ethical Committee for Pediatric Patients (National Ethical Committee for Paediatric Clinical Trials at AIFA) under approval number 113/STDGP0088563.

### 2.2 Data Sources and Measures

The data for this study were obtained from the institutional database of IRCCS Policlinico San Donato, which includes comprehensive clinical records of the medical history of paediatric patients who underwent cardiac surgery [22]. The study includes two distinct cohorts: Cohort A consists of all patients under 18 with CHD who received the PHMB care bundle from April to December 2023, while Cohort B includes patients with CHD who received conventional care from 2019 to 2022 before the PHMB bundle was implemented.

The dataset includes measures such as the source of admission (ward or Intensive Care Unit, ICU), disease classification (categorised as great complexity lesions, moderate complexity lesions or simple lesions) [23] and primary surgical procedure (coded as complete correction of complex heart defects, other specific procedures, reinterventions or valve surgeries) [24]. Additionally, data on previous heart surgeries (yes/no, and if yes, the number of surgeries), previous cardiac catheterisation (yes/no, and if yes, the number of procedures), weight (kg) and age (years) were included. For Cohort A, to ensure adherence, compliance with both Prontoderm wipes (used on the day before surgery) and Prontosan Wound Gel X (applied pre‐dressing) was tracked by healthcare staff and documented as part of institutional protocols. Use of the wipes on any day other than the day before surgery was considered non‐compliant. This detailed tracking supported consistency and adherence to bundle protocols.

Postoperative measures recorded in the dataset include the length of stay in the ICU (days), length of stay in the surgical ward (days), postoperative extracorporeal membrane oxygenation (ECMO, yes, and if yes, the number of days), postoperative open sternum (yes, and if yes, the number of days) and the occurrence of reinterventions during postoperative recovery. Furthermore, the duration of surgical drainage (days) and the presence of SSI were documented, providing a comprehensive overview of patient outcomes and facilitating a robust analysis of the impact of the PHMB care bundle on SSI rates.

### 2.3 Study Population: Inclusion and Exclusion Criteria

The study population comprises paediatric patients with CHD under the age of 18 years who underwent cardiac surgery at IRCCS Policlinico San Donato. The inclusion criteria for this study are as follows: patients must have undergone cardiac surgery within the specified periods, with Cohort A consisting of patients who received the PHMB care bundle from April 2023 to December 2023, and Cohort B including patients who received conventional care from 2019 to 2022. Patients must have complete clinical and anamnestic records available for analysis.

The exclusion criteria included patients who did not undergo sternotomy and/or thoracotomy, as well as those with incomplete clinical records or insufficient documentation verifying adherence to the protocol. Patients with known allergies or sensitivities to PHMB were also excluded. Additionally, patients who exhibited significant deviations from the prescribed antimicrobial procedures—such as non‐adherence to the PHMB care bundle or conventional care protocols—were excluded from the analysis. Records with insufficient documentation regarding the compliance with PHMB care bundle were carefully reviewed and classified as non‐compliant as no information regarding compliance was recorded. Compliance, in this context, is directly related to treatment fidelity, which is a cornerstone of evidence‐based best practices in clinical research [25, 26]. The exclusion of these records was deemed necessary to ensure the integrity of the comparison groups and to avoid potential biases arising from the inclusion of patients whose adherence to the intervention could not be confirmed.

### 2.4 Exposure Groups

The study consists of two exposure groups.

The first group, Cohort A (*n* = 117), includes paediatric patients who received the PHMB care bundle starting from April 2023 to December 2023. This care bundle comprised specific interventions, including an educational program that provided specialised training for healthcare professionals such as nurses, surgeons and anaesthesiologists on SSI prevention. On the day before surgery, patients were treated with Prontoderm wipes, designed for antimicrobial cleansing and MDROs coverage, including MRSA, extended‐spectrum beta‐lactamase‐producing bacteria and vancomycin‐resistant enterococci [27]. This cleansing was generally assisted by healthcare staff to ensure thorough application, followed by the provision of clean gowns and linens. The wipes, formulated with surfactants and PHMB, create a sustained antimicrobial barrier without rinsing. In the operating room, Prontosan Wound Gel X was applied by trained healthcare professionals before dressing the surgical wound [28]. This gel cleansed and moisturised the wound, optimising the healing environment and reducing biofilm risk. Application adjustments were made for delayed closures as necessary. Standard dressings were consistently used for all patients, and no topical negative pressure dressings were applied, even for high‐risk patients [29].

The second group, Cohort B (*n* = 801), comprised paediatric patients who received conventional care from September 2020 to March 2023, before the implementation of the PHMB care bundle. Conventional care involved standard preoperative cleansing with soap and water, routine perioperative antiseptic preparation using agents such as povidone‐iodine or chlorhexidine and standard postoperative wound care without the specialised PHMB interventions described for Cohort A. No structured educational programs specifically targeting SSI prevention, nor targeted antimicrobial protocols involving PHMB, were included. This conventional care group served as a control to assess the impact of the specialised PHMB care bundle.

### 2.5 Outcomes and Treatment Information

The primary outcome of this study is the occurrence rates of SSIs in paediatric patients undergoing cardiac surgery [7, 8].SSIs were defined following CDC guidelines as infections occurring at or near the surgical incision within 30 days post‐operation or up to 1 year if an implant was used [14]. Data on SSIs were collected prospectively through continuous surveillance by trained personnel, capturing infections detected at both primary admission and readmission. Post‐discharge surveillance was undertaken to identify SSIs occurring outside the hospital setting. Secondary outcomes include the length of hospital stay, readmission rates, duration of antibiotic therapy and reoperation needs due to SSI‐related complications.

Treatment for SSIs followed established clinical guidelines, with superficial incisional SSIs managed through antiseptic cleaning and targeted oral antibiotics [18]. Superficial incisional SSIs were managed with antiseptic cleaning, daily dressing changes and targeted oral antibiotics against common pathogens like *Staphylococcus aureus* . For deep incisional SSIs, treatment involved surgical debridement and intravenous antibiotics based on culture results. Negative pressure wound therapy (NPWT) was an option for promoting healing in complex cases. Organ/space SSIs required surgical drainage of infected collections, supported by targeted intravenous antibiotic therapy. Supportive care, including ICU management, was available for systemic complications as needed, aligning with standard paediatric care protocols.

### 2.6 Propensity Score Matching

Propensity score matching was employed to ensure a balanced comparison between the two cohorts and control for potential confounding variables. The propensity score model incorporated covariates selected for clinical relevance and also modelled with restricted cubic splines to capture non‐linear relationships. Covariates showing an absolute standardised difference (ASD) higher than 0.1 and/or high clinical potential contribution in determining the outcome were retained in the propensity score model to ensure adequate adjustment for confounding [30]. Based on these criteria, the logistic regression model used for propensity score calculation is as follows:

(1) $$\begin{array}{c} \text{logit}\left(P\left(\text{Cohort}=A\right)\right)=\beta_{0}+\beta_{1}\cdot \mathrm{ns}\left(\mathrm{Age},\mathrm{df}=3\right)\\ \;+\beta_{2}\cdot \mathrm{ns}\left(\text{Weight},\mathrm{df}=3\right)\\ \;+\beta_{3}\cdot \mathrm{ns}\left(\text{Length}\_\mathrm{ICU},\mathrm{df}=3\right)\\ \;+\beta_{4}\cdot \mathrm{ns}\left(\text{Drainage}\_\text{days},\mathrm{df}=3\right)\\ \;+\beta_{5}\cdot \text{Origin}+\beta_{6}\cdot \text{Previoussurgery}\\ \;+\beta_{7}\cdot \text{Previouscat}\_\mathrm{lab}\\ \;+\beta_{8}\cdot \text{Opensternum}+\beta_{9}\cdot \text{ECMO} \end{array}$$

In this model, the term logit(*P*(Cohort = A)) represents the log odds of being in Cohort A. The intercept term, *β*_0_, indicates the baseline log odds of a patient being in Cohort A when all other variables are at their reference levels. The covariate *β*_1_ · ns(Age, df = 3) captures the non‐linear effect of age on the probability of being in Cohort A, modelled using restricted cubic splines with 3° of freedom. Similarly, *β*_2_ · ns(Weight, df = 3) represents the non‐linear effect of weight, *β*_3_ · ns (Length_ICU, df = 3) models the non‐linear effect of the length of stay in the ICU and *β*_4_ · ns(Drainage_days, df = 3) accounts for the non‐linear effect of the number of drainage days. The variable *β*_5_ · Origin assesses the effect of the source of admission, whether the patient was admitted from the ward or the ICU. The term *β*_6_ · Previoussurgery captures the effect of previous heart surgeries on the likelihood of being in Cohort A and *β*_7_ · Previouscat_lab evaluates the impact of previous cardiac catheterisations. The effect of the postoperative open sternum is represented by *β*_8_ · Opensternum, while *β*_9_ · ECMO examines the influence of postoperative ECMO support.

Matching was performed using a 1:1 nearest‐neighbour method with a 0.05 calliper to ensure close matching, minimising differences in propensity scores between paired individuals. Balance diagnostics confirmed sufficient balance post‐matching, including variance ratio, empirical cumulative distribution function measures and standardised differences.

After calculating the propensity scores, a 1:1 matching procedure was conducted: for each patient in Cohort A, a corresponding patient from Cohort B with a similar propensity score was selected. The matching process was carefully monitored to ensure that the balance between the groups was maintained using several balance diagnostics, including the variance ratio, empirical cumulative distribution function mean, empirical cumulative distribution function maximum and standardised pair distance. This matching aimed to create comparable groups, minimising the influence of confounding variables and allowing for a more accurate assessment of the impact of the PHMB care bundle on the occurrence rates of SSIs. The procedure identified 114 patients in Cohort A and 112 in Cohort B.

### 2.7 Sample Size

The minimum sample size for Cohort A and Cohort B was calculated to ensure the representativity of our cohorts in relation to the study population and to detect a group with a likelihood of representing the infection occurrence rate observed over the past 10 years (from 2013 to 2023) in the setting of the study, which is ~12%. The population size was considered to be ~400 paediatric patients, representing the yearly target population undergoing surgery at IRCCS Policlinico San Donato. Therefore, a precision‐based approach was adopted to determine an appropriate sample size given one proportion in a finite population to adequately reflect the infection occurrence rates by employing the following approach [31]:

(2) $$n=\frac{N\cdot Z^{2}\cdot p\cdot \left(1-p\right)}{\left(N-1\right)\cdot E^{2}+Z^{2}\cdot p\cdot \left(1-p\right)}$$

In this approach, *N* is the population size (~400 paediatric patients), *Z* is the *Z*‐score (e.g., 1.96 for 95% confidence level), *p* is the estimated proportion of the population with the characteristic of interest (12% or 0.12) and *E* is the precision of the estimate (5%). Calculations return 116 patients per cohort.

### 2.8 Statistical Analysis

Data were summarised according to their nature and distribution. A preliminary analysis was conducted to describe the distribution of the data and assess missingness, as outlined in the study protocol. Missing data were to be assessed using multiple methods to evaluate the pattern and mechanism of missingness under the assumption of Missing at Random (MAR) [32]. This included conducting a missingness pattern analysis to determine whether the missing data were related to observed variables and not to unobserved ones. Additionally, a comparison of baseline characteristics between patients with complete and incomplete data was planned to identify potential biases [32]. However, no missing data were identified in the dataset, so these procedures were not required in the final analysis. The three excluded records in the study selection were excluded due to insufficient documentation verifying adherence to the procedure; therefore, the records were excluded by applying the exclusion criteria. Notably, all three excluded cases did not develop SSIs, implying that the exclusion was unrelated to influencing the results concerning SSI outcomes.

Proportions were employed for categorical data, and quantitative data were summarised based on their distribution: normally distributed data with means and standard deviations (SD), while non‐normally distributed data were summarised with medians and interquartile ranges (IQR). Initial comparisons between the two cohorts were performed using ASD for both proportions and quantitative data. For proportions, ASD was calculated as the difference in proportions divided by the pooled standard error. For quantitative data, ASD was calculated as the difference in means divided by the pooled standard deviation. ASD was interpreted as follows: values below 0.1 indicated a negligible imbalance, values between 0.1 and 0.2 indicated a moderate imbalance and values above 0.2 indicated a substantial imbalance [30].

After the propensity score matching procedure, inferential comparisons were performed based on the nature of the variable and its distribution. Exact tests were employed when possible to ensure the robustness of the statistical inferences and in relation to the primary aim. Significance was set with an alpha level of 5%, and *p* values were adjusted for multiple comparisons using the Benjamini–Hochberg procedure to control the likelihood of inflating type I errors. All tests were two‐sided. Statistical analyses were performed in an R environment (R Core Team, 2023) with the following libraries: haven, MatchIt, dplyr, splines, cobalt, ggplot2 and Exact.

## 3 Results

### 3.1 Record Selection

**FIGURE 1:**
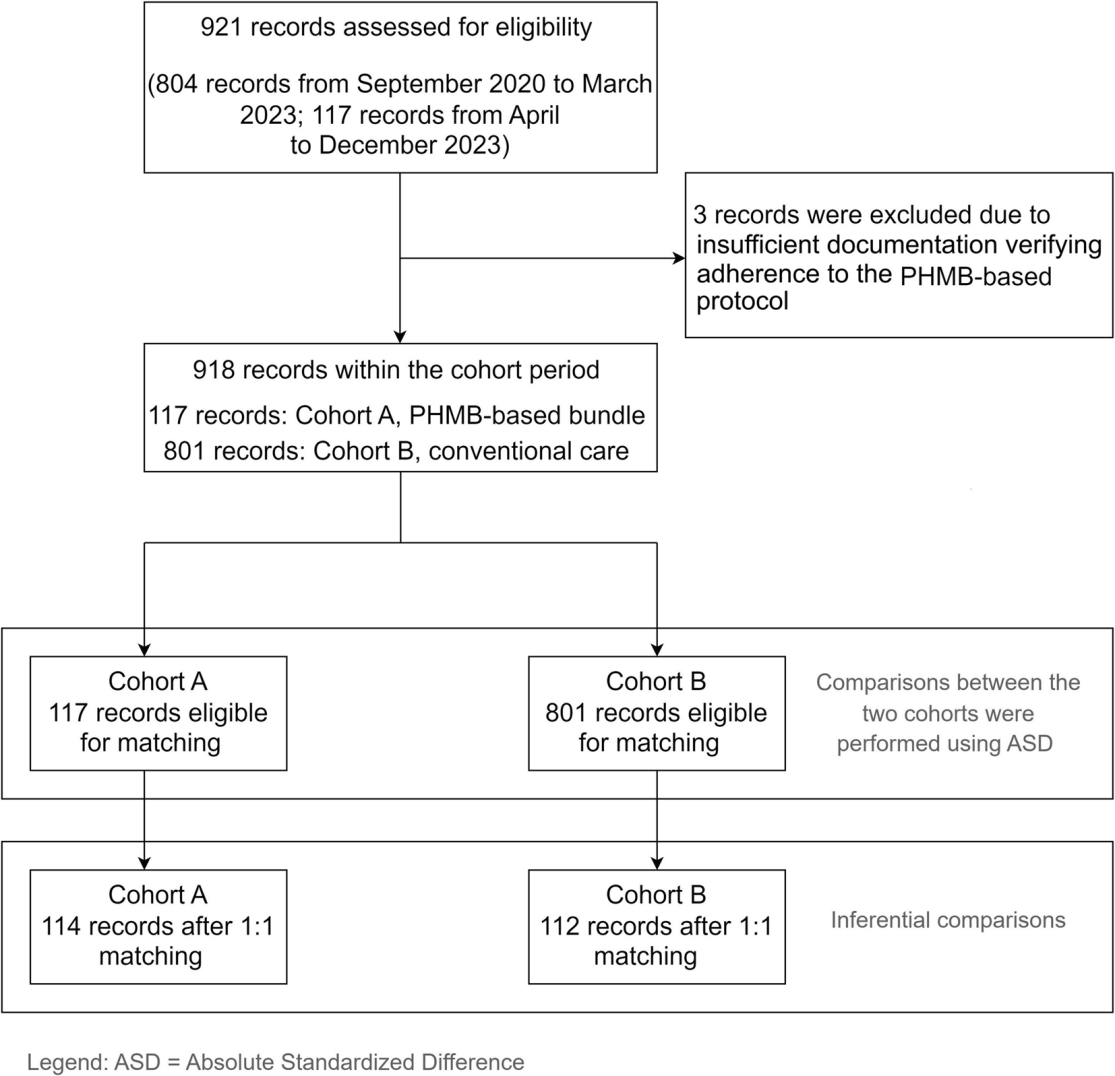
Flow chart of record selection. This diagram illustrates the selection of 921 records assessed for eligibility, including 117 patients exposed to the PHMB‐based care bundle (Cohort A) and 801 patients receiving conventional care (Cohort B). After excluding 3 records due to non‐adherence to the PHMB‐based protocol, 918 records were retained for matching. Following 1:1 propensity score matching, 114 records in Cohort A and 112 in Cohort B were included in the final analysis.

As shown in Figure 1, a total of 921 records were assessed for eligibility, with 804 records from September 2020 to March 2023 and 117 records from April to December 2023. Three records were excluded due to being evaluated as non‐compliant with the PHMB‐based bundle (3 patients with simple lesions, ages 4, 6 and 8; no previous surgeries, no SSI in the follow‐up). This resulted in a final sample of 918 records within the cohort period. Of these, 117 records belonged to Cohort A (PHMB‐based bundle) and 801 records belonged to Cohort B (conventional care). From Cohort A, 117 records were eligible for matching and from Cohort B, 801 records were eligible for matching. After 1:1 matching, Cohort A had 114 records and Cohort B had 112 records.

### 3.2 Characteristics of the Included Cohorts

Table 1 presents the descriptive statistics for the included cohorts before matching. Key baseline differences were observed, notably in the distribution of disease complexity, primary surgical procedures and ICU admission rates. In Cohort A, most patients underwent complete correction of complex heart defects, while Cohort B had a larger proportion of less complex procedures. There were also minor imbalances in previous surgeries, as well as weight and age distributions between the groups, though these differences were controlled for in the matched analysis. Postoperative outcomes showed slight imbalances, with Cohort B experiencing slightly longer lengths of stay in the surgical ward. However, these differences had low ASD values, indicating a limited impact on overall cohort comparability. Detailed values regarding descriptive statistics and ASD scores are provided in Table 1.

**TABLE 1:** Descriptive statistics.

	Cohort A (*N* = 117)		Cohort B (*N* = 801)		Absolute standardised differences
	*n*	%	*n*	%	
Source of admission					
Ward	98	83.8	692	86.4	0.073
ICU	19	16.2	109	13.6	0.073
Disease classification					
Great complexity lesions	33	28.2	254	31.7	0.076
Moderate complexity lesions	67	57.3	502	62.7	0.11
Simple lesions	17	14.5	45	5.6	0.3
Primary surgical procedure					
Complete correction of complex heart defects	100	85.5	365	45.6	0.926
Other specific procedures	3	2.6	33	4.1	0.083
Re‐interventions	3	2.6	21	2.6	0
Valve surgeries	11	9.4	50	6.2	0.119
Previous heart surgeries					
No	82	70.1	569	71	0.02
One previous surgery	23	19.7	138	17.2	0.064
Two previous surgeries	9	7.7	64	8	0.011
Three previous surgeries	2	1.7	25	3.1	0.092
Four previous surgeries	1	0.9	5	0.6	0.035
Previous cardiac catheterisation					
No	98	83.8	680	84.9	0.03
One previous catheterisation	16	13.7	90	11.2	0.076
Two previous catheterisations	3	2.6	21	2.6	0
More than two catheterisations	0	0	10	1.3	0.162
Weight					
kg (median; IQR)	10.49	(4.89–22.00)	10.2	(4.36–23.95)	0.021
Age					
Years (median; IQR)	2.12	(0.3–7.2)	1.9	(0.22–7.59)	0.042
Length of stay in ICU (post‐operative)					
Days (median; IQR)	2	(1–6)	2	(1–6)	0
Length of stay in surgical ward (post‐operative)					
Days (median; IQR)	16	(9–27)	14	(9–23)	0.167
Post‐operative ECMO					
Yes	4	3.6	36	4.5	0.046
Days (median; IQR)	4	(1.75–6.5)	4.5	(3–7)	0.154
Post‐operative open sternum					
Yes	27	23.08	171	21.35	0.043
Days (median; IQR)	3	(2–3.5)	3	(2–4)	0
Reinterventions during post‐operative recovery					
No	107	91.5	778	97.1	0.243
Surgical drainage					
Days (median; IQR)	4	(3–6)	4	(3–6.5)	0.243
SSI					
Yes	2	1.8	28	3.5	0.113
*Pseudomonas aeruginosa*	1	0.9	2	0.2	0.094
*Klebsiella pneumoniae*	1	0.9	8	1.0	0.01
*E. coli*	0	0	4	0.5	0.1
MSSA	0	0	3	0.4	0.004
Acinetobacter	0	0	2	0.2	0.002
*Candida lusitaniae*	0	0	1	0.1	0.045
*Klebsiella aerogenes*	0	0	3	0.4	0.004
MRSE	0	0	5	0.6	0.11

*Note:* Cohort A = patients adopted Polyhexanide Care Bundle (year: 2023); Cohort B = patients operated before the implementation of the Polyhexanide Care Bundle (years: 2020–2022).

Abbreviations: ECMO, extracorporeal membrane oxygenation; ICU, intensive care unit; IQR, interquartile range; MRSE, methicillin‐resistant *Staphylococcus epidermidis* ; MSSA, methicillin‐sensitive *Staphylococcus aureus* ; SSI, surgical site infection.

### 3.3 Propensity Score Matching

**FIGURE 2:**
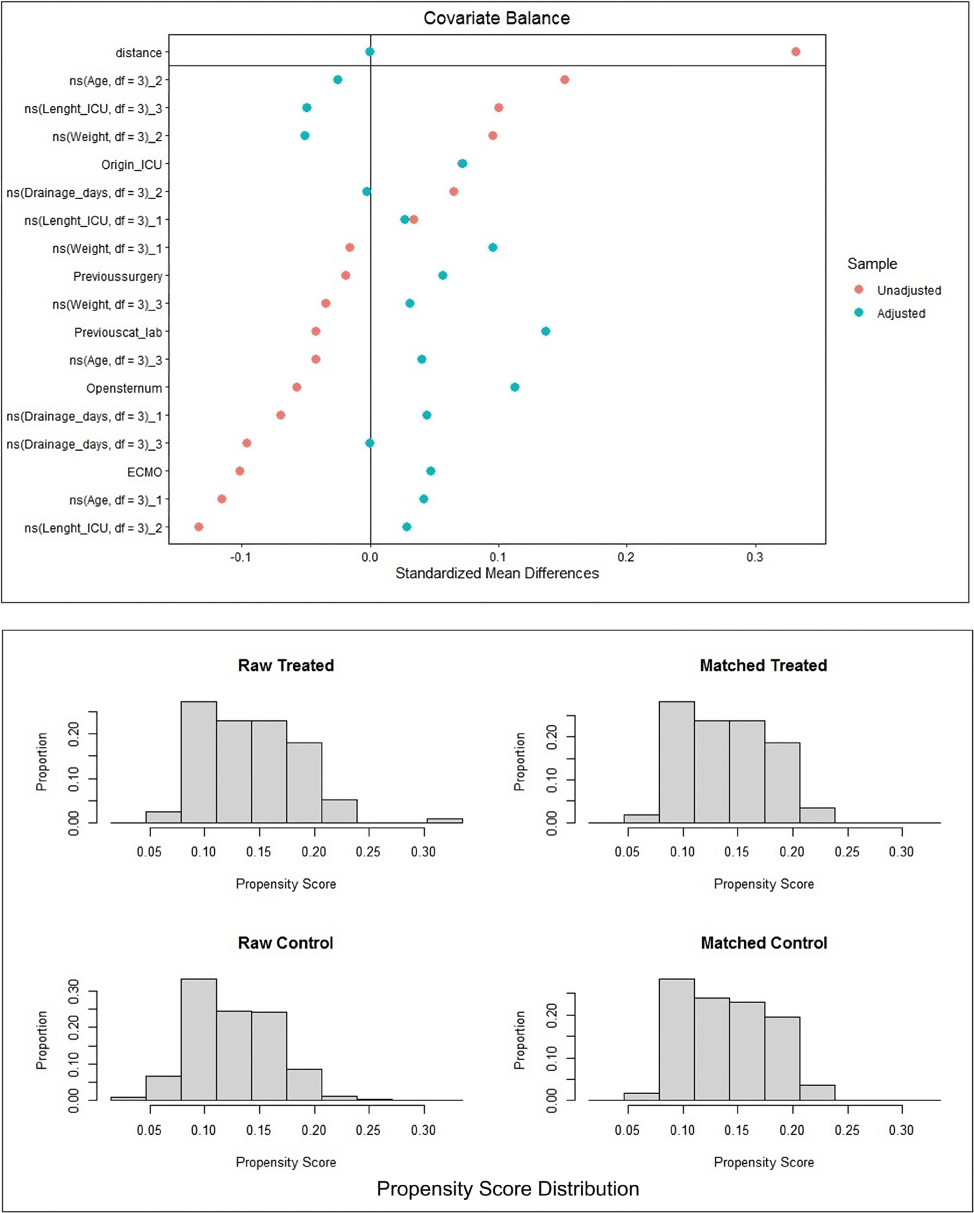
Covariate balance and propensity score distribution. The top panel displays standardised mean differences for each covariate before (red) and after (blue) propensity score matching, demonstrating improved balance post‐matching. The bottom panel shows the distribution of propensity scores in treated and control groups before and after matching, indicating effective alignment of covariate distribution between the two groups.

Figure 2 depicts the covariate balance and propensity score distribution before and after matching. The covariate balance is depicted in the top quadrant of Figure 2, showing the standardised mean differences for each covariate. This visualisation indicates the effectiveness of the matching process in achieving balance between the cohorts. Covariates that were initially imbalanced were brought into closer alignment post‐matching, as evidenced by the reduced standardised mean differences.

The bottom quadrants of Figure 2 display histograms of the propensity scores. The bottom left quadrant shows the distribution of propensity scores for the raw treated group, and the bottom middle left quadrant shows the distribution for the raw control group. These histograms highlight the initial imbalance in the distribution of propensity scores between the groups. The bottom right quadrant shows the distribution for the matched treated group, and the bottom middle right quadrant shows the distribution for the matched control group, illustrating the improved balance achieved through matching.

### 3.4 Comparisons After 1:1 Matching

Table 2 presents the comparisons between the matched cohorts (Cohort A with 114 patients and Cohort B with 112 patients). The source of admission showed no significant difference between the cohorts, with 84.2% of Cohort A and 91.1% of Cohort B being admitted from the ward (*p* = 0.118) and 15.8% of Cohort A and 8.9% of Cohort B being admitted from the ICU. Disease classification was similarly balanced, with great complexity lesions present in 28.1% of Cohort A and 27.7% of Cohort B (*p* = 0.373). Moderate complexity lesions were found in 57.9% of Cohort A and 65.2% of Cohort B, while simple lesions were present in 14% of Cohort A and 7.1% of Cohort B.

**TABLE 2:** Comparisons between matched cohorts.

	Cohort A (*N* = 114)		Cohort B (*N* = 112)		*p*
	*n*	%	*n*	%	
Source of admission					
Ward	96	84.2	102	91.1	0.118
ICU	18	15.8	10	8.9	
Disease classification					
Great complexity lesions	32	28.1	31	27.7	0.373
Moderate complexity lesions	66	57.9	61	65.2	
Simple lesions	16	14	8	7.1	
Primary surgical procedure					
Complete correction of complex heart defects	98	85.96	90	80.36	0.685
Other specific procedures	3	2.63	5	4.46	
Re‐interventions	3	2.63	3	2.68	
Valve surgeries	10	8.77	14	12.50	
Previous heart surgeries					
No	80		79	70.5	0.731
One previous surgery	22		16	14.3	
Two previous surgeries	9		14	12.5	
Three previous surgeries	2		2	1.8	
Four previous surgeries	1		1	0.9	
Previous cardiac catheterisation					
No	95	83.3	93	83	0.793
One previous catheterisation	16	14	15	13.4	
Two previous catheterisations	3	2.6	3	2.7	
More than two catheterisations	0	0	1	0.9	
Weight					
kg (median; IQR)	10.38	(4.8–22)	10.62	(5.51–20.08)	0.912
Age					
Years (median; IQR)	2.1	(0.3–7.2)	2.65	(0.47–6.18)	0.66
Length of stay in ICU (post‐operative)					
Days (median; IQR)	2	(1–6)	2	(1–5)	0.745
Length of stay in surgical ward (post‐operative)					
Days (median; IQR)	16	(8.75–26.25)	13	(8–22.75)	0.256
Post‐operative ECMO					
Yes	3	2.7	3	2.7	0.991
Days (median; IQR)	6	(4–7)	3	(3–3.5)	0.658
Post‐operative open sternum					
Yes	89	78.1	92	82.1	0.549
Days (median; IQR)	3	(2–3)	2	(2–3)	0.401
Reinterventions during post‐operative recovery					
No	105	92.1	110	98.2	0.068
Surgical drainage					
Days (median; IQR)	4	(3–6)	4	(3–6)	0.88
SSI					
Yes	2	1.8	8	7.1	0.048
*Pseudomonas aeruginosa*	1	0.9	0	0	0.556
*Klebsiella pneumoniae*	1	0.9	4	3.6	
Acinetobacter	0	0	1	0.9	
*Klebsiella aerogenes*	0	0	2	1.9	
MRSE	0	0	1	0.9	

*Note:* Cohort A = patients adopted Polyhexanide Care Bundle (year: 2023); Cohort B = patients operated before the implementation of the Polyhexanide Care Bundle (years: 2020–2022).

Abbreviations: ECMO, extracorporeal membrane oxygenation; ICU, intensive care unit; IQR, interquartile range; MRSE, methicillin‐resistant *Staphylococcus epidermidis* ; MSSA, methicillin‐sensitive *Staphylococcus aureus* ; SSI, surgical site infection.

Primary surgical procedures did not differ significantly between the cohorts, with complete correction of complex heart defects performed in 85.96% of Cohort A and 80.36% of Cohort B (*p* = 0.685). Other specific procedures, reinterventions and valve surgeries were similarly distributed across both groups.

Regarding previous heart surgeries, 70.5% of Cohort B had no prior surgeries compared with 79% in Cohort A (*p* = 0.731). The proportions of patients with one, two, three and four previous surgeries were comparable between the cohorts. Previous cardiac catheterisation rates were also similar, with 83.3% of Cohort A and 83% of Cohort B having no prior catheterisations (*p* = 0.793). The distribution of one, two and more than two previous catheterisations showed no significant differences.

The median weight of patients was 10.38 kg (IQR: 4.8–22) in Cohort A and 10.62 kg (IQR: 5.51–20.08) in Cohort B (*p* = 0.912). The median age was 2.1 years (IQR: 0.3–7.2) in Cohort A and 2.65 years (IQR: 0.47–6.18) in Cohort B (*p* = 0.66).

Postoperative lengths of stay in the ICU and surgical ward were also comparable, with a median ICU stay of 2 days (IQR: 1–6) for Cohort A and 2 days (IQR: 1–5) for Cohort B (*p* = 0.745). The median stay in the surgical ward was 16 days (IQR: 8.75–26.25) for Cohort A and 13 days (IQR: 8–22.75) for Cohort B (*p* = 0.256). Postoperative ECMO usage was equal in both cohorts, with 2.7% of patients requiring ECMO in each group (*p* = 0.991) and the median duration was 6 days (IQR: 4–7) in Cohort A and 3 days (IQR: 3–3.5) in Cohort B (*p* = 0.658). Postoperative open sternum rates were also similar, with 78.1% in Cohort A and 82.1% in Cohort B (*p* = 0.549), and a median duration of 3 days (IQR: 2–3) for Cohort A and 2 days (IQR: 2–3) for Cohort B (*p* = 0.401). Reinterventions during postoperative recovery were slightly more frequent in Cohort B, with 92.1% of Cohort A and 98.2% of Cohort B not requiring reinterventions (*p* = 0.068). Surgical drainage durations were identical, with a median of 4 days (IQR: 3–6) in both cohorts (*p* = 0.88).

The occurrence of SSIs was significantly lower in Cohort A, with 1.8% compared with 7.1% in Cohort B (*p* = 0.048). Specific pathogens, including *Pseudomonas aeruginosa* , *Klebsiella pneumoniae* , Acinetobacter, *Klebsiella aerogenes* and MRSE, were identified, but differences between cohorts were not substantial.

## 4 Discussion

The primary novelty of this study lies in its comprehensive evaluation of the PHMB care bundle's impact on the occurrence rates of SSIs in paediatric and neonatal cardiac surgery, and to our knowledge, this is the first study in this regard. Our findings indicate a statistically significant reduction trend in SSIs among patients receiving the PHMB care bundle compared with those receiving conventional care. This study is particularly noteworthy as it addresses the critical gap in paediatric‐specific infection prevention protocols, highlighting the relationship of a structured, evidence‐based approach tailored to this patient population with SSI rates. The results underscore the potential of the PHMB care bundle to enhance clinical outcomes and reduce healthcare burdens associated with postoperative infections in the macro‐framework of complex interventions and antimicrobial stewardship activities [33, 34].

The PHMB care bundle represents a complex intervention due to its multifaceted nature, involving several components that work synergistically to prevent SSIs. While PHMB was a key component of the bundle, the observed reduction in SSI rates likely reflects the combined impact of all bundle components, including educational programs, standardised preoperative and postoperative practices and antimicrobial stewardship measures, rather than the isolated effect of PHMB. According to Craig et al. [35], complex interventions are characterised by multiple interacting components, variability in outcomes and the need for tailored implementation strategies. The PHMB care bundle includes educational programs for healthcare providers, preoperative and postoperative antimicrobial treatments and consistent application of best practices. Each of these elements targets different aspects of infection control, from reducing microbial load on the skin to maintaining a sterile surgical environment and promoting proper wound care. The complexity arises from the interaction between these components and the need for precise execution and adherence by the healthcare team. This comprehensive approach targets the direct prevention of SSIs and aligns with broader antimicrobial stewardship goals by minimising unnecessary antibiotic use and promoting optimal infection control practices [36].

While our study highlights the specific impact of the PHMB care bundle, it is essential to consider that broader advancements in infection prevention practices may have contributed to the observed reductions in SSI rates. The baseline SSI rate in our setting was estimated at 12%, yet both cohorts in our study showed lower infection rates. This discrepancy could reflect an overall improvement in infection control protocols and increased awareness of surgical hygiene practices, which may enhance outcomes regardless of the specific intervention. These broader improvements underscore the importance of implementing comprehensive care bundles within the context of robust infection control policies. They also indicate that while the PHMB bundle demonstrated additional benefits, its relationships with SSI rates were likely complemented by existing infection prevention measures. More precisely, it is likely that the observed associations in our study reflect the interplay of each component of the PHMB care bundle rather than the impact of the antimicrobial agent alone. This synergy among educational programs, preoperative and postoperative antimicrobial treatments and the consistent application of best practices highlights the importance of a comprehensive and coordinated approach to infection control [34, 36].

Our findings align with previous research that underscores the relationships between PHMB and preventing infections across various surgical settings [19, 37]. A recent meta‐analysis has shown that PHMB, with its broad‐spectrum antimicrobial properties, significantly reduces the incidence of postoperative infections, even if it has never been tested in paediatric cardiac surgery [19]. Thus, this study extends the existing literature by focusing specifically on paediatric and neonatal cardiac surgery, a context often underrepresented in infection control research [22]. Previous studies have predominantly addressed adult populations or generalised surgical settings, making our targeted approach a valuable addition to the body of knowledge [37].

The implications of this study for clinical practice are substantial. Implementing antimicrobial care bundles in paediatric cardiac surgery settings could lead to a marked decrease in SSIs, and PHMB shows evidence of its applicability in this regard. In general, our results advocate for the broader adoption of structured, evidence‐based care bundles, emphasising the importance of targeted interventions in paediatric care. Training and educating healthcare teams on the effective use of PHMB care bundles should be prioritised to ensure consistent and effective application.

Future research should focus on further validating these findings across diverse paediatric populations and surgical contexts. Longitudinal studies examining the long‐term outcomes of patients treated with the PHMB care bundle are warranted to assess sustained benefits and potential late‐onset complications. Additionally, exploring the cost‐effectiveness of PHMB care bundles compared with other antimicrobial strategies could provide valuable insights for healthcare policymakers. Research should also aim to refine and optimise the components of the care bundle, identifying the most impactful elements and exploring potential synergies with other infection control measures.

This study's results are intended not as raw comparisons between Cohort A and Cohort B but as analyses based on the matched cohorts, where propensity score matching was used to create balance across covariates. In this regard, this study has several limitations that have to be acknowledged in order to properly interpret the study's results. While robustly managed through propensity score matching, the retrospective design inherently limits the ability to establish causality, as unmeasured confounding factors may still influence observed baseline differences. Additionally, the propensity score matching process reduced sample size, which may affect the statistical power and generalisability of the results. In this regard, the further exclusion of three cases in our study was necessary due to the absence of critical information regarding their compliance with the PHMB care bundle protocol. According to our study protocol, cases with insufficient documentation on compliance were treated as non‐compliant and excluded from the analysis. This approach was adopted to maintain treatment fidelity, which is essential for ensuring reliable comparisons between the PHMB care bundle and conventional care groups [25, 26]. Without documented compliance, it would not have been possible to reliably categorise these patients, thereby introducing potential bias. However, this exclusion criterion introduces a limitation: by reducing the overall sample size, the statistical power of our findings may have been impacted, which could limit the generalisability of the results. Furthermore, if the excluded cases differ systematically from those included (e.g., if they were patients less likely to adhere to protocols or had different clinical outcomes), this could introduce an unmeasured bias, potentially affecting the study's conclusions.

Another limitation pertains to the inability to isolate the specific impact of antibiotics included as part of the PHMB care bundle. Antibiotic use likely contributed significantly to the observed reduction in SSI rates, making it difficult to determine the relative contribution of PHMB compared with antibiotics or other components of the bundle. Future studies should aim to disentangle these effects to better understand the role of antibiotics in combination with PHMB within a care bundle. In addition, the study's single‐centre setting may also limit its applicability to other settings. Another potential limitation is related to compliance with the care bundle; while adherence was monitored, variations in implementation may have introduced a bias in the observed relationship between the PHMB bundle and SSI rates. Moreover, despite our matching process, the potential for residual confounding cannot be entirely excluded. However, the study's strengths lie in its targeted focus on a vulnerable population, the comprehensive data collection based on real‐world data, and the robust statistical methods employed.

## 5 Conclusions

Our retrospective study reports trends toward a potential reduction in paediatric SSIs with the use of PHMB‐based care bundles. These findings provide a foundation for future research to further elucidate the role of PHMB in infection prevention bundles and underscore the need for prospective, controlled study designs. The PHMB care bundle, as a complex intervention, integrates educational programs, antimicrobial treatments and best practices, addressing multiple aspects of infection control. The observed synergy among these components supports the importance of a comprehensive approach to potentially improve infection outcomes. Future research should focus on validating these results in diverse paediatric populations and surgical contexts, assessing long‐term benefits and exploring the cost‐effectiveness of the PHMB care bundle.

## Conflicts of Interest

The authors declare that they have no known competing financial interests or personal relationships that could have appeared to influence the work reported in this article. Authors G.C. and G.G. are married to each other, but this personal relationship has not influenced the conduct or reporting of the work described in this article.

## Data Availability

The data supporting this study's findings are available from the corresponding author upon reasonable request.
